# Cryptic transmission of SARS-CoV-2 in Washington state

**DOI:** 10.1126/science.abc0523

**Published:** 2020-09-10

**Authors:** Trevor Bedford, Alexander L. Greninger, Pavitra Roychoudhury, Lea M. Starita, Michael Famulare, Meei-Li Huang, Arun Nalla, Gregory Pepper, Adam Reinhardt, Hong Xie, Lasata Shrestha, Truong N. Nguyen, Amanda Adler, Elisabeth Brandstetter, Shari Cho, Danielle Giroux, Peter D. Han, Kairsten Fay, Chris D. Frazar, Misja Ilcisin, Kirsten Lacombe, Jover Lee, Anahita Kiavand, Matthew Richardson, Thomas R. Sibley, Melissa Truong, Caitlin R. Wolf, Deborah A. Nickerson, Mark J. Rieder, Janet A. Englund, The Seattle Flu Study Investigators, James Hadfield, Emma B. Hodcroft, John Huddleston, Louise H. Moncla, Nicola F. Müller, Richard A. Neher, Xianding Deng, Wei Gu, Scot Federman, Charles Chiu, Jeffrey S. Duchin, Romesh Gautom, Geoff Melly, Brian Hiatt, Philip Dykema, Scott Lindquist, Krista Queen, Ying Tao, Anna Uehara, Suxiang Tong, Duncan MacCannell, Gregory L. Armstrong, Geoffrey S. Baird, Helen Y. Chu, Jay Shendure, Keith R. Jerome

**Affiliations:** 1 Vaccine and Infectious Disease Division, Fred Hutchinson Cancer Research Center, Seattle, WA, USA.; 2 Brotman Baty Institute for Precision Medicine, Seattle, WA, USA.; 3 Department of Genome Sciences, University of Washington, Seattle, WA, USA.; 4 Department of Laboratory Medicine and Pathology, University of Washington, Seattle, WA, USA.; 5 Institute for Disease Modeling, Bellevue, WA, USA.; 6 Division of Infectious Disease, Seattle Children’s Hospital, Seattle, WA, USA.; 7 Department of Medicine, Division of Allergy and Infectious Diseases, University of Washington, Seattle, WA, USA.; 8 Department of Pediatrics, University of Washington, Seattle, WA, USA.; 9 Biozentrum, University of Basel, Basel, Switzerland.; 10 Swiss Institute of Bioinformatics, Lausanne, Switzerland.; 11 Molecular and Cellular Biology Program, University of Washington, Seattle, WA, USA.; 12 Department of Laboratory Medicine, University of California San Francisco, San Francisco, CA, USA.; 13 Public Health — Seattle & King County, Seattle, WA, USA.; 14 Washington State Department of Health, Shoreline, WA, USA.; 15 Division of Viral Diseases, National Center for Immunization and Respiratory Diseases, Centers for Disease Control and Prevention, Atlanta, GA, USA.; 16 Office of Advanced Molecular Detection, National Center for Emerging and Zoonotic Infectious Diseases, Centers for Disease Control and Prevention, Atlanta, GA, USA.; 17 Howard Hughes Medical Institute, Seattle, WA, USA.

## Abstract

After its emergence in Wuhan, China, in late November or early December 2019, the severe acute respiratory syndrome coronavirus 2 (SARS-CoV-2) virus rapidly spread globally. Genome sequencing of SARS-CoV-2 allows the reconstruction of its transmission history, although this is contingent on sampling. We analyzed 453 SARS-CoV-2 genomes collected between 20 February and 15 March 2020 from infected patients in Washington state in the United States. We find that most SARS-CoV-2 infections sampled during this time derive from a single introduction in late January or early February 2020, which subsequently spread locally before active community surveillance was implemented.

## A series of unfortunate events

The history of how severe acute respiratory syndrome coronavirus 2 (SARS-CoV-2) spread around the planet has been far from clear. Several narratives have been propagated by social media and, in some cases, national policies were forged in response. Now that many thousands of virus sequences are available, two studies analyzed some of the key early events in the spread of SARS-CoV-2. Bedford *et al.* found that the virus arrived in Washington state in late January or early February. The viral genome from the first case detected had mutations similar to those found in Chinese samples and rapidly spread and dominated subsequent undetected community transmission. The other viruses detected had origins in Europe. Worobey *et al.* found that early introductions into Germany and the west coast of the United States were extinguished by vigorous public health efforts, but these successes were largely unrecognized. Unfortunately, several major travel events occurred in February, including repatriations from China, with lax public health follow-up. Serial, independent introductions triggered the major outbreaks in the United States and Europe that still hold us in the grip of control measures.

*Science*, this issue p. 571, p. 564

## Teaser

The first case of SARS-CoV-2 in Washington state occurred at the end of January and, by going undetected, sparked community transmission.

The novel coronavirus, referred to alternately as severe acute respiratory syndrome coronavirus 2 (SARS-CoV-2) (*1*) or human coronavirus 2019 (hCoV-19) (*2*), emerged in Wuhan, Hubei, China, in late November or early December 2019 (*3*). As of 18 May 2020, there have been >4 million confirmed cases of coronavirus disease 2019 (COVID-19)—the disease caused by SARS-CoV-2—that have resulted in >300,000 deaths (*4*). After its initial emergence in China, travel-associated cases with travel histories related to Wuhan appeared in other parts of the world (*5*). The first confirmed case in the United States was travel associated and was detected in Snohomish County, Washington state, on 19 January 2020. Until 27 February 2020, the U.S. Centers for Disease Control and Prevention (CDC) guidance recommended prioritizing testing for COVID-19 on persons with direct travel history from an affected area or with exposure to a known case. Cases of respiratory disease with no known risk factors were not routinely tested. In the 6 weeks between 19 January and 27 February, 59 confirmed cases were reported in the United States (*6*), all outside of Washington state and with either direct travel history or exposure to a known, confirmed case. On 28 February 2020, a community case was identified in Snohomish County (*7*). One month later, on 25 March, as a result of increased testing and ongoing transmission, Washington state reported 2580 confirmed cases and 132 deaths (*8*). Here, we report on the putative history of early community transmission in Washington state as revealed by genomic epidemiology. We conclude that SARS-CoV-2 was circulating for several weeks undetected by the surveillance apparatus in Washington state from late January to early February 2020.

**Fig. 1:**
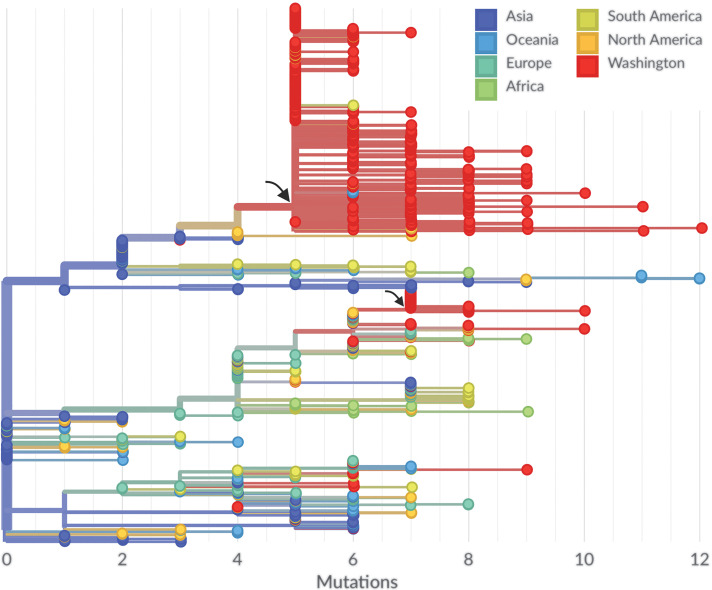
Maximum-likelihood phylogeny of 455 SARS-CoV-2 viruses collected from Washington state on a background of 493 globally collected viruses. Viruses collected from Washington state are shown as red circles. Tips and branches are colored on the basis of location, branch lengths are proportional to the number of mutations along a branch, and the *x* axis is labeled with the number of substitutions relative to the root of the phylogeny—here equivalent to basal Wuhan outbreak viruses. The clustering of related viruses indicates community transmission after an introduction event. Branch locations are estimated on the basis of a discrete traits model. We observe a single introduction leading to a large outbreak clade of 384 sampled viruses from Washington state (marked by the larger arrow), and we observe a second introduction leading to a smaller outbreak clade of 39 viruses (marked by the smaller arrow). An interactive version of this figure is available at https://nextstrain.org/community/blab/ncov-cryptic-transmission/introductions.

Although publicly available SARS-CoV-2 genomes (*9*, *10*) are not sampled in strict proportion to the burden of infections through time and across geography, their genetic relationships can still shed light on underlying patterns of spread. SARS-CoV-2 genomes sampled between December 2019 and 15 March 2020 appear to be closely related, with between 0 and 12 mutations relative to a common ancestor estimated to exist in Wuhan between late November and early December 2019 (Fig. 1). This pattern is consistent with a reported rate of molecular evolution of ~0.8 × 10^−3^ substitutions per site per year or approximately two substitutions per genome per month (*3*). After its initial zoonotic emergence in Wuhan (*11*), SARS-CoV-2 viral genomes began to accumulate substitutions and spread from Wuhan to other regions in the world (*3*). During December 2019, the Wuhan outbreak was too small to seed many introductions outside of China, but by January 2020, it had grown large enough to begin seeding cases elsewhere (*12*).

Sequencing of viruses from the Washington state outbreak began on 28 February 2020 and has continued since then. We analyzed the sequences of 455 SARS-CoV-2 viruses from this outbreak collected between 19 January and 15 March 2020 (Fig. 1). Virus sequences from Washington state are closely related to those from viruses collected elsewhere. Clusters of closely related viruses indicate separate introduction events followed by local spread. The majority (*n* = 384; 84%) of these viruses fall into a closely related clade (marked by the larger arrow in Fig. 1), and these viruses have single-nucleotide polymorphisms (SNPs) C8782T, C17747T, A17858G, C18060T, and T28144C relative to the basal virus at the root of the phylogeny, which is equivalent to the reference virus Wuhan/Hu-1/2019. This clade derives from viruses circulating in China (Fig. 1, in blue), is closely related to viruses sampled in British Columbia (Fig. 1, in orange), and is labeled as Pangolin lineage A.1 (*13*). Going forward, we refer to this clade as the Washington state outbreak clade. Other viruses (*n* = 39; 9%) fall into a separate, smaller clade (marked by the smaller arrow in Fig. 1) and derive from viruses circulating in Europe. The remaining 33 viruses (7%) from Washington state are distributed across the phylogeny. Thus, we conclude that most early cases descend from a single introduction event followed by local amplification.

**Fig. 2:**
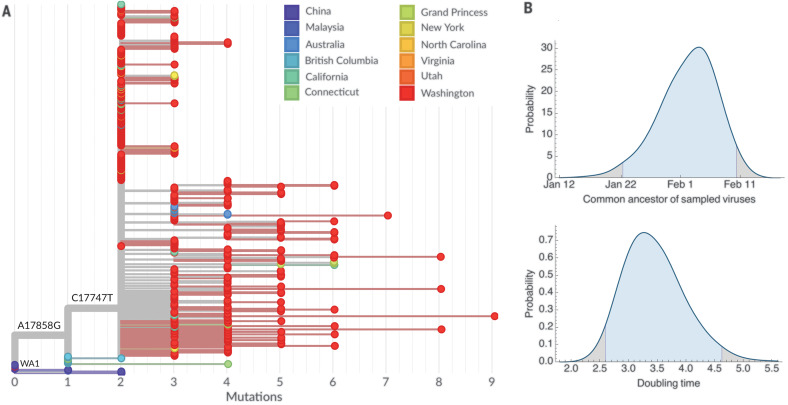
Maximum-likelihood phylogeny of the Washington state outbreak clade and immediately ancestral variants containing 448 SARS-CoV-2 viruses and Bayesian estimates of the date of the outbreak common ancestor and outbreak doubling time. (**A**) Maximum-likelihood phylogeny. Tips are colored on the basis of location, branch lengths are proportional to the number of mutations between viruses, and the *x* axis is labeled with the number of substitutions relative to the root of the phylogeny—here equivalent to the WA1 haplotype. This comb-like phylogenetic structure of the Washington state outbreak clade is consistent with rapid exponential growth of the virus population. An interactive version of this figure is available at https://nextstrain.org/community/blab/ncov-cryptic-transmission/wa-clade. (**B**) Highest posterior density estimates for the date of the common ancestor of viruses from the Washington state outbreak clade (top) as well as the doubling time in days of the growth of this clade (bottom).

The Washington state outbreak clade has a highly comb-like structure (Fig. 2A), which is indicative of rapid exponential growth (*14*). This clade has a C17747T change relative to viruses sampled in British Columbia and a A17858G change relative to viruses sampled in Fujian, Chongqing, Hangzhou, and Guangdong. Given the limited and nonrepresentative sampling of viruses for sequencing, along with the rate of molecular evolution, it is difficult to make detailed assessments of geographic origins. However, we can be confident that this clade represents an introduction from China followed by local spread within the United States and Canada. British Columbia may have been the entry point or the location at which the first virus was sampled.

We analyzed the Washington state outbreak clade in a coalescent analysis to estimate evolutionary dynamics. Here, we assume a prior on evolutionary rate based on analysis of viruses sampled globally between December 2019 and July 2020 (see materials and methods). This analysis uses the degree and pattern of genetic diversity of sampled genomes to estimate the date of a common ancestor and the exponential growth rate of the virus population. We obtained a median estimate for the date of the clade’s common ancestor of 2 February 2020, with a 95% Bayesian credible interval of 22 January to 10 February 2020 (Fig. 2B). We note that the initiation of a transmission chain may slightly predate the common ancestor belonging to this chain in sampled viruses, as initial transmission events after introduction may not result in branching of the transmission tree. We calculated a rate of exponential growth from the coalescent analysis for this clade and found a median doubling time of 3.4 days, with a 95% Bayesian credible interval of 2.6 to 4.6 days (Fig. 2B).

In addition to the 384 viruses from Washington state identified in the Washington state outbreak clade, we observed 12 viruses from elsewhere, including from California, Connecticut, Minnesota, New York, North Carolina, Virginia, Utah, Australia, and the Grand Princess cruise ship (Fig. 2A). Viruses from outside Washington state nest within the diversity found in Washington state. In the case of the Grand Princess, the genetic relationship among these viruses is consistent with a single introduction onto the cruise ship of the basal outbreak variant—having C17747T and A17858G changes—and subsequent transmission and evolution on the ship.

The first confirmed case recorded in the United States was a travel-associated case from an individual returning from Wuhan on 15 January 2020, who presented for care at an outpatient clinic in Snohomish County on 19 January 2020 and tested positive (*15*). This infection is recorded as strain USA/WA1/2020 (referred to here as WA1 and annotated in Fig. 2A), and it appears to be closely related to viruses from infections in China (Fujian, Hangzhou, and Guangdong provinces). Viruses from the Washington state outbreak clade group together as direct descendants of WA1 and its identical relatives (Fig. 2A). This tree structure is consistent with the WA1 strain transmitting locally after arrival into the United States. The rarity of the C8782T, T28144C, and C18060T mutations—characteristic of WA1—in viruses sampled from China (found in 6 of 224 or 3% of sequenced viruses) indicates that this is a parsimonious explanation for the origin of the Washington state outbreak clade. However, because the evolution rate for SARS-CoV-2 (one mutation per ~15 days) is slower than the transmission rate (one transmission event every 4 to 8 days) (*16*, *17*), it is possible that WA1 sits on a side branch of the underlying transmission tree even if it appears as a direct ancestor in the maximum-likelihood tree. The fact that viruses sampled from British Columbia interdigitate between WA1 and the Washington state outbreak clade indicates that this clade may have been introduced into North America by a closely related infection to—but one distinct from—WA1 (Fig. 2A). Additionally, it remains possible that multiple viruses with the basal Washington state outbreak clade genotype were introduced, which resulted in the local amplification of this clade; however, this is markedly less likely than a single introduction of the virus.

**Fig. 3:**
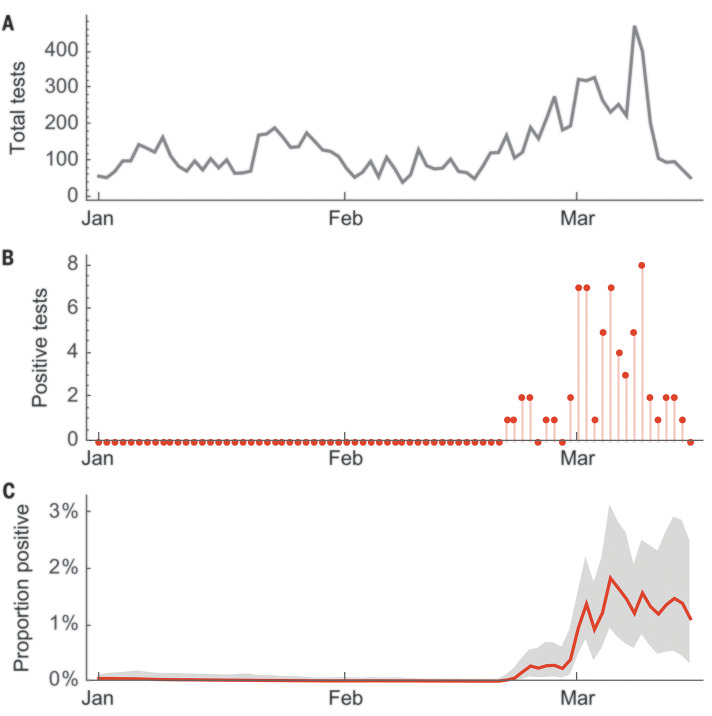
Acute respiratory samples tested for SARS-CoV-2 collected as part of the Seattle Flu Study between 1 January and 15 March 2020. (**A**) Total samples tested per day. In total, 10,382 samples collected between 1 January and 15 March were tested. (**B**) Number of samples testing positive per day. (**C**) Estimated proportion positive using a sequential Monte Carlo model to provide day-to-day smoothing. The solid red line is the mean estimate of proportion positive, and the gray shaded region is the 95% credible interval. All dates are those of sample collection, not dates of testing.

Given that community transmission was first detected on 28 February 2020 from a transmission chain originating between 22 January and 10 February 2020, we sought to address community prevalence during this period. Here, we analyzed 10,382 acute respiratory specimens collected as part of the Seattle Flu Study between 1 January and 15 March 2020 (Fig. 3A). These specimens represented a mix of residual samples collected as part of routine clinical testing and samples collected as part of prospective community enrollment of individuals with acute respiratory illness. In total, 5270 samples collected between 1 January and 20 February tested negative. The first positive sample was collected on 21 February (Fig. 3B). From 21 February to 15 March, of 5112 samples collected, 65 samples tested positive. On 1 March, a sequential Monte Carlo procedure estimated the proportion of acute respiratory specimens positive for SARS-CoV-2 as 1.1% with a 95% credible interval of 0.5 to 2.0% (Fig. 3C). It is challenging to directly convert this value into population prevalence of SARS-CoV-2; however, U.S. Health Weather data show a 4.5% prevalence of influenza-like illness on 1 March (*18*), from which we estimated a 0.05% population prevalence of SARS-CoV-2.

In January and February 2020, screening for SARS-CoV-2 in the United States was directed at travelers with fever, cough, and shortness of breath, with the point of origin broadening as new outbreaks were identified but continuing to solely specify travel to China up until 24 February 2020 (*19*, *20*). Our analysis indicates that at least one clade of SARS-CoV-2 had been circulating in the Seattle area for 3 to 6 weeks by the time the virus was first detected in a nontraveler on 28 Feb 2020. By then, variants within this clade constituted the majority of confirmed infections in the region (384 of 455; 84%). Several factors could have contributed to the delayed detection of presumptive community spread, including limited testing among nontravelers or the presence of asymptomatic or mild illnesses.

Both the WA1 strain sampled in Snohomish County, Washington, on 19 January as well as viruses sampled from British Columbia in early March appear to be phylogenetically ancestral to viruses from the Washington state outbreak clade (Fig. 2A), which suggests a possible route of introduction. However, in both of these cases, a lack of comprehensive geographic sampling makes it difficult to rely on phylogenetic structure for transmission inference. Viruses sampled from British Columbia may derive from local spread after a direct introduction event, or they may be offshoots of an introduction elsewhere that subsequently spread to British Columbia. Refining the time and geographic origin of the introduction into Washington state will require a combination of earlier samples and samples from other geographic locations. Other states in the United States have shown different genetic histories from that seen in Washington state, with most SARS-CoV-2 sequences from New York (*21*) and Connecticut (*22*) clustering with European lineages, which indicates repeated introductions from Europe. We also observed a second cluster of Washington state viruses related to a later introduction from Europe.

Our results highlight the critical need for widespread surveillance for community transmission of SARS-CoV-2 throughout the United States and the rest of the world, even after the current pandemic is brought under control. The broad spectrum of disease severity (*23*) makes such surveillance challenging (*24*). The combination of traditional public health surveillance and genomic epidemiology can provide actionable insights, as happened in this instance: Upon sequencing the initial community case on 29 February 2020, results were immediately shared with national, state, and local public health agencies, which resulted in the rapid rollout of social distancing policies as Seattle and Washington state came to grips with the extent of existing COVID-19 spread. The confirmation of local transmission in Seattle prompted a change in testing criteria to emphasize individuals with no travel history. From 29 February onward, genomic data were immediately posted to the GISAID EpiCoV sequence database (*9*, *10*) and analyzed alongside other public SARS-CoV-2 genomes by means of the Nextstrain online platform (*25*) to provide immediate and public situational awareness. We see the combination of community surveillance, genomic analysis, and public real-time sharing of results as a pathway to empower infectious disease surveillance systems.

## Supplementary Materials

science.sciencemag.org/content/370/6516/571/suppl/DC1 Materials and Methods Fig. S1 Seattle Flu Study Investigators List References (*27*–*40*) MDAR Reproducibility Checklist Data S1

## References

[1] A. E. Gorbalenya, S. C. Baker, R. S. Baric, R. J. de Groot, C. Drosten, A. A. Gulyaeva, B. L. Haagmans, C. Lauber, A. M. Leontovich, B. W. Neuman, D. Penzar, S. Perlman, L. L. M. Poon, D. Samborskiy, I. A. Sidorov, I. Sola, J. Ziebuhr, Severe acute respiratory syndrome-related coronavirus: The species and its viruses – a statement of the Coronavirus Study Group. bioRxiv 2020.02.07.937862 [Preprint]. 11 February 2020. 10.1101/2020.02.07.937862. 10.1101/2020.02.07.937862

[2] Jiang S., Shi Z., Shu Y., Song J., Gao G. F., Tan W., Guo D., A distinct name is needed for the new coronavirus. Lancet 395, 949 (2020). 10.1016/S0140-6736(20)30419-0 32087125 PMC7124603

[3] A. Rambaut, Phylogenetic analysis of nCoV-2019 genomes (Virological, 2020); https://virological.org/t/phylodynamic-analysis-176-genomes-6-mar-2020/356.

[4] World Health Organization (WHO), “Coronavirus disease 2019 (COVID-19): Situation report – 55” (WHO, 2020); www.who.int/docs/default-source/coronaviruse/situation-reports/20200315-sitrep-55-covid-19.pdf?sfvrsn=33daa5cb_8.

[5] F. Schlosser, B. F. Maier, O. Baranov, D. Brockmann, C. Jongen, A. Zachariae, A. Rose, Coronavirus COVID-19 Global Risk Assessment, Event Horizon - COVID-19 (2020); http://rocs.hu-berlin.de/corona/.

[6] World Health Organization (WHO), “Coronavirus disease 2019 (COVID-19): Situation report – 38” (WHO, 2020); www.who.int/docs/default-source/coronaviruse/situation-reports/20200227-sitrep-38-covid-19.pdf?sfvrsn=2db7a09b_4.

[7] K. Bray, “Coronavirus update: Addressing questions about a presumptive positive case in an adolescent” (Snohomish Health District Public Health Essentials, 2020); www.snohd.org/Blog.aspx?IID=13.

[8] Washington State Department of Health, “2019 Novel Coronavirus Outbreak (COVID-19)” (2020); www.doh.wa.gov/Emergencies/Coronavirus.

[9] Shu Y., McCauley J., GISAID: Global initiative on sharing all influenza data - from vision to reality. Euro Surveill. 22, 30494 (2017). 10.2807/1560-7917.ES.2017.22.13.30494 28382917 PMC5388101

[10] Elbe S., Buckland-Merrett G., Data, disease and diplomacy: GISAID’s innovative contribution to global health. Glob Chall 1, 33–46 (2017). 10.1002/gch2.1018 31565258 PMC6607375

[11] Andersen K. G., Rambaut A., Lipkin W. I., Holmes E. C., Garry R. F., The proximal origin of SARS-CoV-2. Nat. Med. 26, 450–452 (2020). 10.1038/s41591-020-0820-9 32284615 PMC7095063

[12] N. Imai, I. Dorigatti, A. Cori, C. Donnelly, S. Riley, N. M. Ferguson, “Report 2: Estimating the potential total number of novel Coronavirus cases in Wuhan City, China” (Imperial College London, 2020); http://hdl.handle.net/10044/1/77150.

[13] Rambaut A., Holmes E. C., O’Toole Á., Hill V., McCrone J. T., Ruis C., du Plessis L., Pybus O. G., A dynamic nomenclature proposal for SARS-CoV-2 lineages to assist genomic epidemiology. Nat. Microbiol. 10.1038/s41564-020-0770-5 (2020). 10.1038/s41564-020-0770-5 32669681 PMC7610519

[14] Volz E. M., Koelle K., Bedford T., Viral phylodynamics. PLOS Comput. Biol. 9, e1002947 (2013). 10.1371/journal.pcbi.1002947 23555203 PMC3605911

[15] Holshue M. L., DeBolt C., Lindquist S., Lofy K. H., Wiesman J., Bruce H., Spitters C., Ericson K., Wilkerson S., Tural A., Diaz G., Cohn A., Fox L., Patel A., Gerber S. I., Kim L., Tong S., Lu X., Lindstrom S., Pallansch M. A., Weldon W. C., Biggs H. M., Uyeki T. M., Pillai S. K., Washington State 2019-nCoV Case Investigation Team, First Case of 2019 Novel Coronavirus in the United States. N. Engl. J. Med. 382, 929–936 (2020). 10.1056/NEJMoa2001191 32004427 PMC7092802

[16] Li Q., Guan X., Wu P., Wang X., Zhou L., Tong Y., Ren R., Leung K. S. M., Lau E. H. Y., Wong J. Y., Xing X., Xiang N., Wu Y., Li C., Chen Q., Li D., Liu T., Zhao J., Liu M., Tu W., Chen C., Jin L., Yang R., Wang Q., Zhou S., Wang R., Liu H., Luo Y., Liu Y., Shao G., Li H., Tao Z., Yang Y., Deng Z., Liu B., Ma Z., Zhang Y., Shi G., Lam T. T. Y., Wu J. T., Gao G. F., Cowling B. J., Yang B., Leung G. M., Feng Z., Early Transmission Dynamics in Wuhan, China, of Novel Coronavirus-Infected Pneumonia. N. Engl. J. Med. 382, 1199–1207 (2020). 10.1056/NEJMoa2001316 31995857 PMC7121484

[17] Nishiura H., Linton N. M., Akhmetzhanov A. R., Serial interval of novel coronavirus (COVID-19) infections. Int. J. Infect. Dis. 93, 284–286 (2020). 10.1016/j.ijid.2020.02.060 32145466 PMC7128842

[18] Miller A. C., Singh I., Koehler E., Polgreen P. M., A Smartphone-Driven Thermometer Application for Real-time Population- and Individual-Level Influenza Surveillance. Clin. Infect. Dis. 67, 388–397 (2018). 10.1093/cid/ciy073 29432526

[19] U.S. Centers for Disease Control and Prevention (CDC), “Update and Interim Guidance on Outbreak of 2019 Novel Coronavirus (2019-nCoV)” (CDC Health Alert Network, 2020); https://emergency.cdc.gov/han/han00427.asp.

[20] U.S. Centers for Disease Control and Prevention (CDC), “Criteria to Guide Evaluation of Persons Under Investigation (PUI) for 2019-nCoV” (CDC, 2020); https://web.archive.org/web/20200222215422/https://www.cdc.gov/coronavirus/2019-ncov/hcp/clinical-criteria.html.

[21] Gonzalez-Reiche A. S., Hernandez M. M., Sullivan M. J., Ciferri B., Alshammary H., Obla A., Fabre S., Kleiner G., Polanco J., Khan Z., Alburquerque B., van de Guchte A., Dutta J., Francoeur N., Melo B. S., Oussenko I., Deikus G., Soto J., Sridhar S. H., Wang Y.-C., Twyman K., Kasarskis A., Altman D. R., Smith M., Sebra R., Aberg J., Krammer F., Garcia-Sarstre A., Luksza M., Patel G., Paniz-Mondolfi A., Gitman M., Sordillo E. M., Simon V., van Bakel H., Introductions and early spread of SARS-CoV-2 in the New York City area. Science 369, 297–301 (2020). 10.1126/science.abc1917 32471856 PMC7259823

[22] Fauver J. R., Petrone M. E., Hodcroft E. B., Shioda K., Ehrlich H. Y., Watts A. G., Vogels C. B. F., Brito A. F., Alpert T., Muyombwe A., Razeq J., Downing R., Cheemarla N. R., Wyllie A. L., Kalinich C. C., Ott I. M., Loman J. N. J., Neugebauer K. M., Greninger A. L., Jerome K. R., Roychoundhury P., Xie H., Shrestha L., Huang M.-L., Pitzer V. E., Iwasaki A., . B.Omer S. B., Khan K., Bogoch I. I., Martinello R. A., Foxman E. F., Landry M. L., Neher R. A., Ko A. I., Grubaugh N. D., Coast-to-coast spread of SARS-CoV-2 during the early epidemic in the United States.. Cell 181, 990–996.e5 (2020). 10.1016/j.cell.2020.04.021 32386545 PMC7204677

[23] Guan W.-J., Ni Z.-Y., Hu Y., Liang W.-H., Ou C.-Q., He J.-X., Liu L., Shan H., Lei C.-L., Hui D. S. C., Du B., Li L.-J., Zeng G., Yuen K.-Y., Chen R.-C., Tang C.-L., Wang T., Chen P.-Y., Xiang J., Li S.-Y., Wang J.-L., Liang Z.-J., Peng Y.-X., Wei L., Liu Y., Hu Y.-H., Peng P., Wang J.-M., Liu J.-Y., Chen Z., Li G., Zheng Z.-J., Qiu S.-Q., Luo J., Ye C.-J., Zhu S.-Y., Zhong N.-S., China Medical Treatment Expert Group for Covid-19, Clinical Characteristics of Coronavirus Disease 2019 in China. N. Engl. J. Med. 382, 1708–1720 (2020). 10.1056/NEJMoa2002032 32109013 PMC7092819

[24] Li R., Pei S., Chen B., Song Y., Zhang T., Yang W., Shaman J., Substantial undocumented infection facilitates the rapid dissemination of novel coronavirus (SARS-CoV-2). Science 368, 489–493 (2020). 10.1126/science.abb3221 32179701 PMC7164387

[25] Hadfield J., Megill C., Bell S. M., Huddleston J., Potter B., Callender C., Sagulenko P., Bedford T., Neher R. A., Nextstrain: Real-time tracking of pathogen evolution. Bioinformatics 34, 4121–4123 (2018). 10.1093/bioinformatics/bty407 29790939 PMC6247931

[26] T. Bedford, blab/ncov-cryptic-transmission: Release 2020-05-31, version 2020-05-31, Zenodo (2020); 10.5281/zenodo.3871089. 10.5281/zenodo.3871089

[27] Chu H. Y., Boeckh M., Englund J. A., Famulare M., Lutz B. R., Nickerson D. A., Rieder M., Starita L., Thompson M., Shendure J., Bedford T., Adler A., Brandstetter E., Bosua J., Frazar C. D., Han P. D., Gulati R., Hadfield J., Huang S. C., Jackson M. L., Kiavand A., Kimball L. E., Lacombe K., Logue J., Lyon V., Sibley T. R., Zigman Suchsland M. L., Wolf C. R., LB21. The Seattle Flu Study: A Community-Based Study of Influenza. Open Forum Infect. Dis. 6, S1002 (2019). 10.1093/ofid/ofz415.2504

[28] H. Y. Chu, M. Boeckh, J. A. Englund, M. Famulare, B. R. Lutz, D. A. Nickerson, M. J. Rieder, L. M. Starita, A. Adler, E. Brandstetter, C. D. Frazar, P. D. Han, R. K. Gularti, J. Hadfield, M. L. Jackson, A. Kiavand, L. E. Kimball, K. Lacombe, J. Logue, V. Lyon, K. L. Newman, T. R. Sibley, M. L. Zigman Suschsland, C. Wolf, J. Shendure, T. Bedford, The Seattle Flu Study: a multi-arm community-based prospective study protocol for assessing influenza prevalence, transmission, and genomic epidemiology. medRxiv 2020.03.02.20029595 [Preprint]. 6 March 2020. . 10.1101/2020.03.02.20029595

[29] Greninger A. L., Zerr D. M., Qin X., Adler A. L., Sampoleo R., Kuypers J. M., Englund J. A., Jerome K. R., Rapid Metagenomic Next-Generation Sequencing during an Investigation of Hospital-Acquired Human Parainfluenza Virus 3 Infections. J. Clin. Microbiol. 55, 177–182 (2016). 10.1128/JCM.01881-16 27795347 PMC5228228

[30] Greninger A. L., Knudsen G. M., Roychoudhury P., Hanson D. J., Sedlak R. H., Xie H., Guan J., Nguyen T., Peddu V., Boeckh M., Huang M.-L., Cook L., Depledge D. P., Zerr D. M., Koelle D. M., Gantt S., Yoshikawa T., Caserta M., Hill J. A., Jerome K. R., Comparative genomic, transcriptomic, and proteomic reannotation of human herpesvirus 6. BMC Genomics 19, 204 (2018). 10.1186/s12864-018-4604-2 29554870 PMC5859498

[31] Greninger A. L., Roychoudhury P., Xie H., Casto A., Cent A., Pepper G., Koelle D. M., Huang M.-L., Wald A., Johnston C., Jerome K. R., Ultrasensitive Capture of Human Herpes Simplex Virus Genomes Directly from Clinical Samples Reveals Extraordinarily Limited Evolution in Cell Culture. MSphere 3, e00283-18 (2018). 10.1128/mSphereDirect.00283-18 29898986 PMC6001610

[32] Bankevich A., Nurk S., Antipov D., Gurevich A. A., Dvorkin M., Kulikov A. S., Lesin V. M., Nikolenko S. I., Pham S., Prjibelski A. D., Pyshkin A. V., Sirotkin A. V., Vyahhi N., Tesler G., Alekseyev M. A., Pevzner P. A., SPAdes: A new genome assembly algorithm and its applications to single-cell sequencing. J. Comput. Biol. 19, 455–477 (2012). 10.1089/cmb.2012.0021 22506599 PMC3342519

[33] M. Vasimuddin, S. Misra, H. Li, S. Aluru, “Efficient architecture-aware acceleration of BWA-MEM for multicore systems,” in *2019 IEEE International Parallel and Distributed Processing Symposium (IPDPS)* (IEEE, 2019), pp. 314–324.

[34] Seemann T., Prokka: Rapid prokaryotic genome annotation. Bioinformatics 30, 2068–2069 (2014). 10.1093/bioinformatics/btu153 24642063

[35] Katoh K., Standley D. M., MAFFT multiple sequence alignment software version 7: Improvements in performance and usability. Mol. Biol. Evol. 30, 772–780 (2013). 10.1093/molbev/mst010 23329690 PMC3603318

[36] Nguyen L.-T., Schmidt H. A., von Haeseler A., Minh B. Q., IQ-TREE: A fast and effective stochastic algorithm for estimating maximum-likelihood phylogenies. Mol. Biol. Evol. 32, 268–274 (2015). 10.1093/molbev/msu300 25371430 PMC4271533

[37] Suchard M. A., Lemey P., Baele G., Ayres D. L., Drummond A. J., Rambaut A., Bayesian phylogenetic and phylodynamic data integration using BEAST 1.10. Virus Evol. 4, vey016 (2018). 10.1093/ve/vey016 29942656 PMC6007674

[38] Hasegawa M., Kishino H., Yano T., Dating of the human-ape splitting by a molecular clock of mitochondrial DNA. J. Mol. Evol. 22, 160–174 (1985). 10.1007/BF02101694 3934395

[39] A. Doucet, N. de Freitas, N. Gordon, in *Sequential Monte Carlo Methods in Practice. Statistics for Engineering and Information Science*, A. Doucet, N. de Freitas, N. Gordon, Eds. (Springer, 2001), pp. 3–14.

[40] Gordon N. J., Salmond D. J., Smith A. F. M., Novel approach to nonlinear/non-Gaussian Bayesian state estimation. IEE Proc. F, Radar Signal Process. 140, 107–113 (1993). 10.1049/ip-f-2.1993.0015

